# Metabolic modeling of a chronic wound biofilm consortium predicts spatial partitioning of bacterial species

**DOI:** 10.1186/s12918-016-0334-8

**Published:** 2016-09-07

**Authors:** Poonam Phalak, Jin Chen, Ross P. Carlson, Michael A. Henson

**Affiliations:** 1 Department of Chemical Engineering and Institute for Applied Life Sciences, University of Massachusetts, 240 Thatcher Way, Life Science Laboratories Building, Amherst, MA 01003 USA; 2 Department of Chemical and Biological Engineering and Center for Biofilm Engineering, Montana State University, Bozeman, MT 59717 USA

**Keywords:** Metabolic modeling, Biofilms, Wound infections

## Abstract

**Background:**

Chronic wounds are often colonized by consortia comprised of different bacterial species growing as biofilms on a complex mixture of wound exudate. Bacteria growing in biofilms exhibit phenotypes distinct from planktonic growth, often rendering the application of antibacterial compounds ineffective. Computational modeling represents a complementary tool to experimentation for generating fundamental knowledge and developing more effective treatment strategies for chronic wound biofilm consortia.

**Results:**

We developed spatiotemporal models to investigate the multispecies metabolism of a biofilm consortium comprised of two common chronic wound isolates: the aerobe *Pseudomonas aeruginosa* and the facultative anaerobe *Staphylococcus aureus*. By combining genome-scale metabolic reconstructions with partial differential equations for metabolite diffusion, the models were able to provide both temporal and spatial predictions with genome-scale resolution. The models were used to analyze the metabolic differences between single species and two species biofilms and to demonstrate the tendency of the two bacteria to spatially partition in the multispecies biofilm as observed experimentally. Nutrient gradients imposed by supplying glucose at the bottom and oxygen at the top of the biofilm induced spatial partitioning of the two species, with *S. aureus* most concentrated in the anaerobic region and *P. aeruginosa* present only in the aerobic region. The two species system was predicted to support a maximum biofilm thickness much greater than *P. aeruginosa* alone but slightly less than *S. aureus* alone, suggesting an antagonistic metabolic effect of *P. aeruginosa* on *S. aureus*. When each species was allowed to enhance its growth through consumption of secreted metabolic byproducts assuming identical uptake kinetics, the competitiveness of *P. aeruginosa* was further reduced due primarily to the more efficient lactate metabolism of *S. aureus*. Lysis of *S. aureus* by a small molecule inhibitor secreted from *P. aeruginosa* and/or *P. aeruginosa* aerotaxis were predicted to substantially increase *P. aeruginosa* competitiveness in the aerobic region, consistent with in vitro experimental studies.

**Conclusions:**

Our biofilm modeling approach allows the prediction of individual species metabolism and interspecies interactions in both time and space with genome-scale resolution. This study yielded new insights into the multispecies metabolism of a chronic wound biofilm, in particular metabolic factors that may lead to spatial partitioning of the two bacterial species. We believe that *P. aeruginosa* lysis of *S. aureus* combined with nutrient competition is a particularly relevant scenario for which model predictions could be tested experimentally.

**Electronic supplementary material:**

The online version of this article (doi:10.1186/s12918-016-0334-8) contains supplementary material, which is available to authorized users.

## Background

In nature, the majority of bacteria grow as biofilms in mixed consortia that use mutualistic, syntrophic, commensal or antagonistic strategies to compete for and efficiently utilize available nutrients [1–4]. Microbial biofilms are critically important in medical, environmental and engineered biological systems. For example, the human gut microbiome has emerged as a major focus for biomedical research with mounting evidence suggesting unhealthy gut flora biofilms are associated with illnesses including autoimmune diseases, colorectal cancer and inflammatory bowel disease [5–10]. Environmental microbial biofilm consortia form the basis of global nutrient cycles from nitrogen fixation to carbon fluxes [11, 12]. Additionally, the study of natural biofilms has gained in popularity recently due to their efficient organization and ability, through synergistic interactions, to optimize multiple tasks simultaneously like the deconstruction of complex, recalcitrant plant materials into simple sugars. A major goal of current biofuels research is to engineer synthetic microbial communities that mimic these naturally occurring biofilms for biomass conversion to renewable liquid fuels [13]. While foundational to the vast majority of microbial life on the planet, the basic design principles of consortial biofilms are still poorly understood due largely to the complexity of naturally occurring systems [3, 4].

Chronic wounds are defined as a host-pathogen environment that has failed to proceed through a timely healing process. An estimated 2 % of the U.S. population (6 million people) have a non-healing chronic wound with treatment costing more than $25 billion per year [14–16]. Chronic wounds are often colonized by microorganisms growing as biofilms on a complex mixture of wound exudate [17–24]. Microbes residing in biofilms exhibit phenotypes distinct from planktonic growth, making treatment a major challenge. For instance, bacteria in biofilms can tolerate antimicrobial agent concentrations 10,000 times higher than the same microbes grown planktonically [25, 26]. Chronic wounds are typically colonized by consortia comprised of different microbial species [17–19, 27, 28]. Polymicrobial infections have been reported to have elevated mortality rates relative to monocultures [29], and in vivo rabbit model systems demonstrated that consortia prevented wound healing compared to their respective monocultures [24, 30].

The aerobe *Pseudomonas aeruginosa* and the facultative anaerobe *Staphylococcus aureus* are two bacteria commonly isolated from chronic would biofilm infections [19, 23, 27]. The same two bacteria are often key contributors to multispecies infections that occur in the lung mucous of cystic fibrosis patients [31]. *P. aeruginosa* is known to exhibit much lower growth rates than *S. aureus* and other facultative anaerobes in anaerobic environments common in chronic wound and mucoid biofilms [32, 33]. Perhaps partially in response to this metabolic disadvantage, *P. aeruginosa* has evolved a number of mechanisms to enhance its competitiveness in multispecies biofilm communities. The most widely studied mechanism is growth inhibition and lysis of competing bacteria through the secretion of an arsenal of small molecule (e.g. pyocyanin [34]) and protein (e.g. bacteriocins [35]) toxins. The consumption of metabolic byproducts secreted by other bacteria through cross feeding mechanisms also has been proposed to enhance *P. aeruginosa* competitiveness [36]. Another putative mechanism is *P. aeruginosa* chemotaxis towards high oxygen niches (i.e. aerotaxis [37]) where it is metabolically competitive.

Multispecies biofilms are sufficiently complex to preclude detailed understanding through traditional experimental techniques developed for planktonic cultures. A primary challenge is the complex interactions between the biofilm species and the extracellular environment [38]. Most naturally occurring microbial consortia exist in spatially heterogeneous environments that also exhibit temporal variations. The presence of spatial heterogeneity plays an essential role in the evolution and function of microbial species [39–43] and has profound effects on biofilm formation and development [3, 38, 44, 45]. Concentration gradients in key nutrients due to limited diffusion establish metabolic niches within the biofilm that can produce spatial variations in biomass density [46] and additionally spatial partitioning of species in the case of multispecies biofilms [45, 47]. Quantitative understanding of the relationships between spatial and temporal variations in the extracellular environment and community metabolism is critical to systematically analyze and rationally manipulate biofilm consortia. While spatiotemporal metabolic models that account for both spatial and temporal variations in the extracellular environment have been constructed, these models rely on table lookups of precomputed flux balance solutions [48–50] or lattice based descriptions of nutrient diffusion [51, 52].

We recently proposed a general methodology for spatiotemporal metabolic modeling based on combining genome-scale reconstructions with fundamental transport equations that capture the relevant convective [53] and/or diffusional [54] processes. In this paper, we applied this methodology to develop biofilm metabolic models that predict the complex spatiotemporal behavior of a *P. aeruginosa*/*S. aureus* two species system. The biofilm models were formulated by combining genome-scale reconstructions of *P. aeruginosa* and/or *S. aureus* metabolism with uptake kinetics and reaction-diffusion type equations for extracellular substrates and metabolic byproducts. To avoid complications associated with solving a moving boundary problem, the biofilm was assumed to have a fixed thickness over which metabolites diffused and cell growth occurred. Therefore, the models were most appropriate for predicting the metabolism of biofilms of a specified thickness. We developed an effective computational method for solving the biofilm models, which consisted of a set of partial differential equations with mixed boundary conditions constrained by embedded linear programs. The models were used to analyze the metabolic differences between single species and two species chronic wound biofilms and to investigate putative factors that could impact the physiology of the two species biofilm, including nutrient diffusion, metabolite cross-feeding, *P. aeruginosa* motility and *P. aeruginosa* mediated lysis of *S. aureus*.

## Methods

### Model formulation

**Fig. 1:**
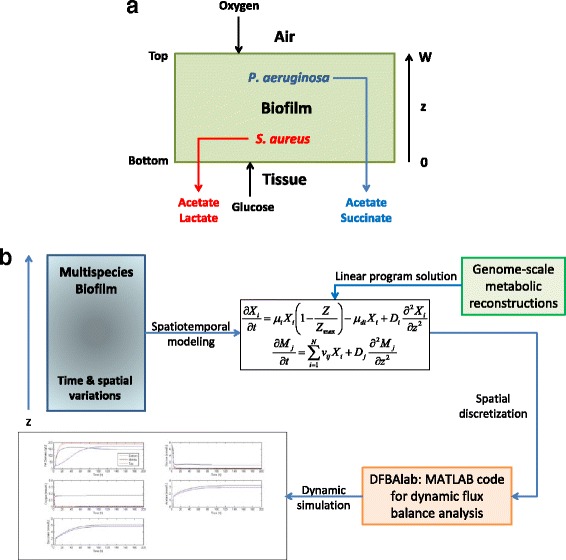
Formulation and solution of the multispecies biofilm metabolic model. **a** Schematic representation of the chronic wound biofilm model of constant thickness *W* with glucose provided at the tissue-biofilm interface (*z* = 0), oxygen supplied at the biofilm-air interface (*z* = *W*) and the metabolic byproducts acetate, succinate and lactate removed at the tissue-biofilm interface. **b** Schematic representation of the biofilm metabolic model solution procedure. The multispecies biofilm with temporal and spatial variations is described by a spatiotemporal model that accounts for the diffusion of nutrients and byproducts. PDEs are written with respect to the bacterial species concentration (*X*_i_) and the metabolite concentrations (*M*_j_) assuming that spatial variations are limited to a single direction *z*. Lexicographic linear program solution of the genome-scale reconstruction of each species is performed to predict the growth rate, nutrient uptake rates and byproduct secretion rates. The PDEs are spatially discretized to yield a large-set of ODEs with embedded LPs that are integrated with the MATLAB code DFBAlab [78] to generate time and spatially resolved predictions

Biofilm models were formulated by combining genome-scale reconstructions of *P. aeruginosa* and/or *S. aureus* metabolism with uptake kinetics for available nutrients and reaction-diffusion type equations for species biomass, supplied substrates and synthesized metabolic byproducts. Single species biofilm models were formulated with either the *P. aeruginosa* or *S. aureus* reconstruction, while the two species model used both reconstructions. Diffusion was assumed to occur only in the axial direction of the biofilm such that spatial variations could be captured with a single variable *z* (Fig. 1a). For simplicity, the biofilm was assumed to have a fixed thickness *W* over which the nutrients diffused and cell growth occurred. Therefore, the models were most appropriate for predicting the metabolism of biofilms of a specified thickness. Both strains were assumed to consume glucose as the primary carbon source [55]. Glucose was supplied at the tissue-biofilm interface at the assumed concentration of the wound exudate, while oxygen was supplied at the biofilm-air interface at a concentration for an aqueous solution in equilibrium with atmospheric oxygen.

The *P. aeruginosa* PA01 iMO1056 reconstruction accounts for 1056 genes, 1030 enzymes, 833 intracellular reactions and 277 exchange reactions [56]. This reconstruction has been shown to provide good agreement with experimentally determined biomass yields for aerobic growth on glucose and anaerobic growth on glucose with nitrate as an electron acceptor. Our preliminary flux balance calculations with a maximum growth objective showed the primary metabolic byproducts to be acetate and L-alanine. The secretion fluxes of other minor byproducts were approximately an order of magnitude less than for acetate and L-alanine. *P. aeruginosa* is known to secrete acetate, lactate and succinate [57], while the secretion of L-alanine has not been reported. To obtain byproduct distributions in better agreement with [57], we constrained the L-alanine secretion flux to zero. This modification resulted in a redirection of flux from L-alanine to succinate with little effect on the secretion fluxes of acetate and minor byproducts. Furthermore, we enforced a minimal non-growth associated ATP maintenance flux of 5 mmol/gDW/h, the same value as in the *S. aureus* reconstruction, to reduce the *P. aeruginosa* anaerobic growth rate for consistency with experimental studies [32]. The iMO1056 reconstruction contained succinate, lactate and acetate uptake fluxes that allowed the investigation of putative cross feeding of metabolic byproducts. Secretion of the small molecule inhibitor pycoyanin was included by adding an exchange flux with an adjustable lower bound that forced pycoyanin synthesis, which was in opposition to growth rate maximization.

The *S. aureus* N315 iMH551 reconstruction accounts for 551 genes, 604 enzymes, 682 intracellular reactions and 92 exchange reactions [58]. This model correctly reproduces byproduct secretion patterns under aerobic conditions with glucose limitation and under anaerobic conditions with glucose excess [59–63]. Our flux calculations showed that the primary byproducts were acetate and lactate. The iMH551 reconstruction contained lactate and acetate uptake fluxes that allowed reassimilation of secreted metabolic byproducts. To explore the possibility of succinate cross feeding, the *S. aureus* model was modified to allow succinate uptake through a putative proton dependent symport mechanism.

Uptake kinetics were specified for the supplied substrates glucose and oxygen as well as for the possible cross-fed metabolites lactate, succinate and acetate. Although both *P. aeruginosa* and *S. aureus* are well known for their ability to perform anaerobic respiration using nitrate as an electron acceptor in place of oxygen, we have neglected the possible role of denitrification in this study. Uptake kinetics were assumed to follow standard Monod expressions of the form,

1 $$ {v}_i={v}_{\max, i}{S}_i\hbox{---} \hbox{---} \hbox{---} \hbox{---} \hbox{---} {K}_{m,i}+{S}_i $$

where *v*_*i*_ is the uptake rate (mmol/gDW/h) of the *i*-th substrate, *S*_*i*_ is the extracellular concentration (mmol//L) of the *i*-th substrate, *v*_*max,i*_ is the maximum uptake rate and *K*_*m,i*_ is the half saturation constant. Equation (1) was used to establish transport bounds on the uptake rates with the actual uptake rates being determined by solution of the intracellular flux balance problem. Both *v*_*max,i*_ and *K*_*m,i*_ were important parameters due to the large nutrient spatial gradients induced by diffusion through the biofilm.

Mass balances on the two species had the form,

2 $$ \begin{array}{ccc}\kern1em \frac{\partial X\left(z,t\right)}{\partial t}={\mu}_XX\left(\mathsf{1}-\frac{Z}{Z_{\max }}\right)-{k}_{dX}X+{k}_A\frac{\partial }{\partial z}\left(X\frac{\partial O}{\partial z}\right)\left(\mathsf{1}-\frac{Z}{Z_{\max }}\right),\kern1em & \kern1em \frac{\partial X\left(\mathsf{0},t\right)}{\partial z}=\mathsf{0},\kern1em & \kern-0.80em \frac{\partial \mathsf{X}\left(\mathsf{W},t\right)}{\partial z}=\mathsf{0}\kern1em \\ {}\kern1em \frac{\partial Y\left(z,t\right)}{\partial t}={\mu}_YY\left(\mathsf{1}-\frac{Z}{Z_{\max }}\right)-{k}_{dY}Y-{k}_LPY,\kern1em & \kern-0.70em \frac{\partial Y\left(\mathsf{0},t\right)}{\partial z}=\mathsf{0},\kern1em & \kern-0.70em \frac{\partial Y\left(W,\mathsf{t}\right)}{\partial z}=\mathsf{0}\kern1em \end{array} $$

where *X* and *Y* are the biomass concentrations (g/L) of *P. aeruginosa* and *S. aureus*, respectively, *Z* = *X* + *Y* is the total biomass concentration, and μ_*X*_ and μ_*Y*_ are the corresponding growth rates (h^−1^) obtained from the flux balance calculations. If local nutrient concentrations became too small to meet the ATP maintenance demand of a species, then the flux balance problem for the species became infeasible at that location. Once an infeasibility was detected, the death rate constant *k*_*dX*_ or *k*_*dY*_ was rapidly increased exponentially from zero to a fixed, non-zero value to simulate that the species would begin to die at that location. This approach ensured that the model equations remained smooth and could be integrated. *P. aeruginosa* has flagella for motility and can aerotaxis towards higher oxygen levels [37]. This capability was captured in the model by including a typical chemotaxis term [64] where *O* is the oxygen concentration (mmol/L) and *k*_*A*_ is the aerotaxis rate constant. Cell growth was restricted to a maximum cell concentration *Z*_*max*_ to account for cell crowding effects within the biofilm. No flux boundary conditions were imposed at the tissue-biofilm (*z* = 0) and biofilm-air (*z* = *W*) interfaces under the assumption that cells could not leave the biofilm via mechanisms such as dispersal.

*P. aeruginosa* secretes pyocyanin and other small molecules that are known to inhibit and lyse of competing bacteria such as *S. aureus* [65]. This lysis mechanism was included in the model through a pyocyanin concentration (*P*, mmol/L) dependent death term with rate constant *k*_*L*_ in the *S. aureus* mass balance in Eq. (2). Pyocyanin synthesis by *P. aeruginosa* and diffusion through the biofilm was captured with the mass balance,

3 $$ \begin{array}{ccc}\hfill \frac{\partial P\left(z,t\right)}{\partial t}={v}_PX+{D}_P\frac{\partial^{\mathsf{2}}P}{\partial {z}^{\mathsf{2}}},\hfill & \hfill -{D}_P\frac{\partial P\left(\mathsf{0},t\right)}{\partial z}={k}_{mP}\left[{P}_b-P\left(0,t\right)\right],\hfill & \hfill \frac{\partial P\left(W,t\right)}{\partial z}\hfill \end{array}=\mathsf{0} $$

where *v*_*P*_ is the specific pyocyanin synthesis rate obtained from the flux balance calculation and *D*_*P*_ is the pyocyanin diffusion coefficient. A no flux boundary condition was imposed at the biofilm-air interface assuming that the pyocyanin was non-volatile. By contrast, a Robin type boundary condition was imposed at the tissue-biofilm interface to describe possibly mass transfer limited removal of pyocyanin, where *k*_*mP*_ is pyocyanin mass transfer coefficient and *P*_*b*_ is the bulk pyocyanin concentration in the tissue.

The glucose and oxygen mass balances were formulated under the assumptions that oxygen gas-liquid mass transfer was fast compared to oxygen uptake and that metabolites had negligible volatilities:

4 $$ \begin{array}{c}\hfill \frac{\partial G\left(z,t\right)}{\mathit{\partial}t}={v}_{GX}X+{v}_{GY}Y+{D}_G\frac{{\mathit{\partial}}^{\mathsf{2}}G}{\mathit{\partial}{z}^{\mathsf{2}}}\hfill \\ {}\hfill \begin{array}{ccc}\hfill -{D}_G\frac{\mathit{\partial}G\left(\mathsf{0},t\right)}{\mathit{\partial}z}={k}_{mG}\left[{G}_b-G\left(\mathsf{0},t\right)\right],\hfill & \hfill \frac{\partial G\left(W,t\right)}{\mathit{\partial}z}=\mathsf{0}\hfill & \hfill \hfill \end{array}\hfill \\ {}\hfill \frac{\mathit{\partial}O\left(z,t\right)}{\mathit{\partial}t}={v}_{OX}X+{v}_{OY}Y+{D}_O\frac{{\mathit{\partial}}^{\mathsf{2}}O}{\mathit{\partial}{z}^{\mathsf{2}}}\hfill \\ {}\hfill \begin{array}{ccc}\hfill -{D}_O\frac{\mathit{\partial}O\left(\mathsf{0},t\right)}{\mathit{\partial}z}={k}_{mO}\left[{O}_b-O\left(\mathsf{0},t\right)\right],\hfill & \hfill -{D}_O\frac{\mathit{\partial}O\left(W,t\right)}{\mathit{\partial}z}={k}_{mO}\left[{O}_a-O\left(W,t\right)\right]\hfill & \hfill \hfill \end{array}\hfill \end{array} $$

where *G* is the glucose concentration (mmol/L), the *P. aeruginosa* uptake fluxes *v*_*GX*_ and *v*_*OX*_ and the *S. aureus* uptake fluxes *v*_*GY*_ and *v*_*OY*_ were obtained from the flux balance calculations, and *D*_*G*_ and *D*_*O*_ are the glucose and oxygen diffusion coefficients. For glucose, a no flux boundary condition was imposed at the biofilm-air interface assuming glucose was not volatile and a Robin type boundary condition was imposed at the tissue-biofilm interface to model possibly mass transfer limited transport of glucose into the biofilm. Here *k*_*mG*_ is the glucose mass transfer coefficient and *G*_*b*_ is the bulk glucose concentration in the wound exudate. For oxygen, Robin type boundary conditions were imposed at both interfaces with oxygen mass transfer coefficient *k*_*mO*_, oxygen concentration *O*_b_ at the tissue-biofilm interface and oxygen concentration *O*_a_ at the biofilm-air interface.

Mass balances on the three primary metabolic byproducts had the form,

5 $$ \begin{array}{c}\hfill \frac{\mathit{\partial}{M}_j\left(z,t\right)}{\mathit{\partial}t}={v}_{M_jX}X+{v}_{M_jY}Y+{D}_{M_j}\frac{{\mathit{\partial}}^{\mathsf{2}}{M}_j}{\mathit{\partial}{z}^{\mathsf{2}}}\hfill \\ {}\hfill \begin{array}{cc}\hfill -{D}_{M_j}\frac{\mathit{\partial}{M}_j\left(\mathsf{0},t\right)}{\mathit{\partial}z}={k}_{m{M}_j}\left[{M}_{j,b}-{M}_j\left(\mathsf{0},t\right)\right],\hfill & \hfill \frac{\mathit{\partial}{M}_j\left(W,t\right)}{\mathit{\partial}z}=\mathsf{0}\hfill \end{array}\hfill \end{array} $$

where *M*_*j*_ is concentration (mmol/L) of the *j*-th byproduct (*A* = acetate, *S* = succinate, *L* = lactate), the secretion (or consumption) fluxes *v*_*MjX*_ and *v*_*MjY*_ were obtained from the flux balance calculations, *D*_*Mj*_ is the diffusion coefficient, *k*_*mMj*_ is the mass transfer coefficient at the tissue-biofilm interface, and *M*_*j,b*_ is the bulk concentration of the wound exudate. No flux boundary conditions were imposed at the biofilm-air interface assuming the byproducts were not volatile, while a Robin type boundary condition was imposed at the tissue-biofilm interface to allow removal of the byproducts from the biofilm.

The biofilm diffusion coefficients were assumed to depend on the total biomass concentration *Z* such that diffusion was reduced in more dense regions of the biofilm [66],

6 $$ {D}_{M_k}={D}_{W{M}_k}\left(\mathsf{1}-\frac{a_{M_k}{Z}^{\mathsf{0.92}}}{\mathsf{11.19}+\mathsf{0.27}{Z}^{\mathsf{0.99}}}\right), $$

where *D*_*Mk*_ is the biofilm diffusion coefficient of the *k*-th species (glucose, oxygen, acetate, succinate, lactate and pyocyanin), *D*_*WMk*_ is the aqueous diffusion coefficients at 37 °C and *a*_*MK*_ is an adjustable parameter. These relations were obtained using a previously proposed empirical equation [66] by adjusting *a*_*MK*_ such that the biofilm diffusion coefficient was equal to the aqueous value when *Z* = 0 and equal to biofilm values reported elsewhere [67] when *Z* = *Z*_*ma*x_. Because pyocyanin has a comparable molecular weight to glucose, the inhibitor diffusion coefficient was chosen to follow the same relation.

### Model parameters

We found a dearth of literature for determining species specific values for the 20 parameters needed to calculate uptake rates with respect to the five possible nutrients (glucose, oxygen, succinate, lactate, acetate). Consequently, the two species were assumed to have the same uptake parameter values. We used representative glucose [68, 69] and oxygen [69, 70] uptake parameter values reported for the model bacterium *Escherichia coli* under the assumption that *P. aeruginosa* and *S. aureus* should have similar values. Because we were not able to find reliable uptake parameter values for succinate, lactate and acetate, the associated *v*_*max*_ and *K*_*m*_ values were assumed to be equal to those for glucose (Table 1). Therefore results focused on differences in metabolic network structure of the two species and not on differences in uptake properties.

**Table 1:** Nominal nutrient uptake parameters

Nutrient	*P. aeruginosa* or *S. aureus*	
	*v*_*max*_ (mmol/gDW/h)	*K*_*m*_ (mmol/L)
Glucose	10	0.5
Oxygen	20	0.003
Succinate	10	0.5
Lactate	10	0.5
Acetate	10	0.5

Other parameter values for the biofilm model were obtained from the literature to the extent possible (Table 2). We utilized a typical biofilm thickness *W* and assumed wound exudate concentrations consistent with published values. The air oxygen concentration *O*_*a*_ was derived from the oxygen content of atmospheric air. *P. aeruginosa* and *S. aureus* cell death were implemented by exponentially increasing the death rate constants from zero to the values listed in Table 2 when local nutrient concentrations were not sufficient to meet ATP maintenance demands. The lower bound on the *P. aeruginosa* pyocyanin synthesis flux *v*_*P*,min_ was tuned such that the average pyocyanin concentration within the biofilm was the same order of magnitude as that observed experimentally in [71]. The *S. aureus* inhibitor-mediated death constant *k*_*L*_ was tuned to achieve reasonable spatial distributions of the two species, which included *P. aeruginosa* dominance in the aerobic region of the biofilm, *S. aureus* dominance in the anaerobic region and a sharp spatial division between the two species [47, 72–74].

**Table 2:** Nominal model parameter values

Parameter	Description	Value	Source
*W*	Biofilm thickness	80 μm	Specified
*G*_*b*_	Bulk glucose concentration	7.5 mmol/L	[75]
*O*_*a*_	Oxygen concentration at the biofilm-air interface	0.21 mmol/L	[103]
*O*_*b*_	Oxygen concentration at the tissue-biofilm interface	0 mmol/L	Specified
*A*_*b*_	Bulk acetate concentration	0 mmol/L	Specified
*S*_*b*_	Bulk succinate concentration	0 mmol/L	Specified
*L*_*b*_	Bulk lactate concentration	1.0 mmol/L	[75]
*P*_*b*_	Bulk pyocyanin concentration	0 mmol/L	Specified
${k}_{dX}$ *,* ${k}_{dY}$	Death rate constants	0–0.01 h^−1^	Calculated
*k*_*L*_	Pyocyanin-associated death rate constant	0.4 mmol/gDW/h	Specified
*k*_*dP*_	Pyocyanin flux bound	0.1 L/mmol/h	Specified
*Z*_*max*_	Maximum biomass concentration	200 g/L	[46]
*km*_*G*_ *, km*_*A*_ *, km*_*S*_, *km*_*L*_ *, km*_*P*_	Mass transfer coefficients for glucose, acetate, succinate, lactate and pyocyanin	2.0 × 10^−4^ cm/s	Specified
*D*_*WG*_	Aqueous diffusion coefficient for glucose	9.4 × 10^−6^ cm^2^/s	[67]
*D*_*WO*_	Aqueous diffusion coefficient for oxygen	26.8 × 10^−6^ cm^2^/s	[67]
*D*_*WA*_	Aqueous diffusion coefficient for acetate	16.2 × 10^−6^ cm^2^/s	[67]
*D*_*WS*_	Aqueous diffusion coefficient for succinate	12.6 × 10^−6^ cm^2^/s	[67]
*D*_*WL*_	Aqueous diffusion coefficient for lactate	12.1 × 10^−6^ cm^2^/s	[104]
*D*_*WP*_	Aqueous diffusion coefficient for pyocyanin	7.2 × 10^−6^ cm^2^/s	Specified
*a*_*G*_, *a*_*S*_, *a*_*L*_, *a*_*P*_	Adjustable parameter for glucose, succinate, lactate and pyocyanin in Eq. (6)	0.33	Fitted
*a*_*O*_	Adjustable parameter for oxygen in Eq. (6)	0.19	Fitted
*a*_*A*_	Adjustable parameter for acetate in Eq. (6)	0.36	Fitted
*X*_*0*_ *, Y*_*0*_	Initial biomass concentrations	1 g/L	Specified
*k*_*A*_	Aerotaxis rate constant	5 × 10^−8^ cm^2.^ L/mmol. s	Specified
*km*_*O*_	Oxygen mass transfer coefficient	2.0 × 10^−2^ cm/s	Specified

The maximum achievable biomass concentration *Z*_*max*_ was chosen to be within the large range of published values [46]. We established reasonable metabolite concentrations within the biofilm by adjusting a single mass transfer coefficient for glucose, acetate, succinate, lactate and pyocyanin such that their average concentrations were the same order of magnitude as those observed experimentally in [71] and [75]. The *P. aeruginosa* aerotaxis rate constant *k*_*A*_ was chosen such that *P. aeruginosa* was dominant in the aerobic region of the biofilm and a sharp spatial division between the two species was established as the biofilm matured towards a steady-state condition [43, 47, 76]. Initial conditions for each simulation were generated by first running a simulation with each species biomass concentration constrained to be 1 g/L and capturing the resulting steady-state solution. These initial conditions reflected a newly developed, nearly spatially homogeneous biofilm with small cell densities, high nutrient levels and low byproduct concentrations.

### Model solution

The two species biofilm model consisted of a set of partial differential equations (PDEs) with mixed boundary conditions and embedded linear programs (LPs). The efficient and stable solution of such models is a challenging problem at the forefront of microbial metabolic modeling [77]. As described in our previous publications [53, 54], we pursued a spatial discretization approach based on converting the PDEs into a large set of ordinary differential equations (ODEs) in time with embedded LPs (Fig. 1b). The spatial domain [0, *W*] was discretized using *N* = 50 node points at which the diffusion terms in Eqs. (2) – (5) were discretized using central difference approximations with second-order accuracy. The specified boundary conditions were incorporated into the central difference approximations at the boundary node points. This procedure yielded a set of 8 ODEs at each node point for the local concentrations of *P. aeruginosa* and *S. aureus* biomass, glucose, oxygen, acetate, succinate, lactate and pyocyanin.

This ODE system was solved using DFBAlab [78], a MATLAB tool that explicitly addresses problems associated with LP alternative optima and possible infeasibilities [79]. DFBAlab employs a lexicographic optimization strategy in which a series of LP problems are sequentially solved to ensure the determination of unique exchange fluxes necessary for a well-defined dynamic system. Each LP is solved subject to constraints that the previous objectives are equal to their optimal values, with the required number of LPs equal to the number of exchange fluxes. We specified the lexicographic optimization objectives to reflect known or anticipated physiology of the two species biofilm community (Table 3). We found that reordering these objectives had no noticeable effect on simulation results. Each node point was represented by 8 ODEs for the local species and metabolite concentrations and 12 LPs for lexicographic optimization. We employed 50 node points such that the discretized biofilm model consisted of 400 ODEs and 600 LPs.

**Table 3:** Lexicographic objective functions

Number	Species	Direction	Objective	Reason
1	PA	Maximize	Growth rate	Assumed primary objective
	SA		Growth rate	
2	PA	Minimize	Acetate secretion flux	Minimize byproduct synthesis
	SA		Acetate secretion flux	
3	PA	Minimize	Succinate secretion flux	Minimize byproduct synthesis
	SA		Lactate secretion flux	
4	PA	Maximize	Glucose uptake flux	Maximize nutrient consumption
	SA			
5	PA	Maximize	Oxygen uptake flux	Maximize nutrient consumption
	SA			
6	PA	Maximize	Lactate uptake flux	Maximize consumption of putative cross-fed metabolite
	SA		Succinate uptake flux	

All simulations were performed with MATLAB 8.5 (R2015a) using DFAlab, the stiff MATLAB integrator ode15s for dynamic flux balance model solution and Gurobi 6.0 for linear program solution. A typical 1000-h dynamic simulation for determining a steady-state solution required about 25 min running on an ASUS computer with Intel Core i7-960 processor and 24 GB RAM. As compared to alternative computational methods for spatiotemporal metabolic modeling based on table lookups of precomputed FBA solutions combined with integration of the PDEs on a coarse spatial grid [48–50] and real-time FBA solution combined with lattice-based descriptions of metabolite diffusion [51, 52], we believe our approach offers several important advantages including the use of DFBAlab, the ability to directly embed LPs within the discretized ODEs, and the flexibility to solve the ODE-LP system using stiff integrators with variable step size and error control.

## Results

### Flux balance analysis of single species metabolism

Flux balance analysis (FBA) was performed on the single species, genome-scale reconstructions to investigate *P. aeruginosa* and *S. aureus* growth rates in different metabolic niches consistent with the bottom, middle and top of the biofilms. More specifically, FBA was used to predict growth rates at three different glucose and oxygen uptake rates. We also performed FBA at three different acetate and lactate uptake rates to investigate the effect of byproduct uptake on growth rates. The growth rate of *S. aureus* was predicted to equal or exceed that of *P. aeruginosa* for all combinations of glucose and oxygen uptake rates, with the difference most pronounced under anaerobic conditions expected at the bottom of the biofilm (Table 4). No growth was predicted for either species under anaerobic, low glucose conditions which may prevail in mature biofilms of sufficient thickness. When acetate was used as the carbon source, *P. aeruginosa* was predicted to have low growth rates that depended only on the acetate uptake rate until oxygen became limiting. *S. aureus* was not able to grow on acetate at any oxygenation level, as has been observed experimentally [80]. As compared to glucose, the absolute growth rates and differences between species growth rates was relatively small for growth on lactate. Collectively, these results suggest that *S. aureus* has a distinct growth rate advantage over *P. aeruginosa* for all conditions anticipated in the simulated two species biofilm.

**Table 4:**
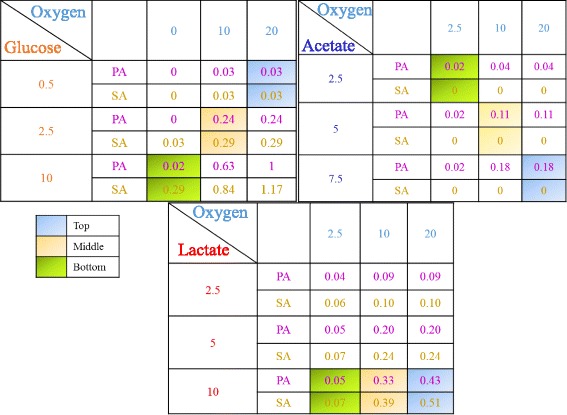
Flux balance analysis of *P. aeruginosa* (PA) and *S. aureus* (SA) single species metabolism. Growth rates (h^−1^) are shown for different combinations of substrate uptake rates (mmol/gDW/h)

Representative uptake rates at the top, middle and bottom of a typical simulated two species biofilm are colored coded

### Metabolism of single species biofilms

Dynamic simulations were performed for single species biofilms consisting of only *P. aeruginosa* or *S. aureus* with glucose and oxygen as the only substrates. Simulations were performed over a time window of 1000 h to capture a steady-state solution, although near steady-state behavior was typically established in <100 h. For each species, biofilms of different thicknesses were simulated to determine the maximum thickness *W*_max_ that could be sustained under the given environmental conditions. If the biomass concentration dropped below 10 g/L (5 % of the maximum value *Z*_max_ = 200 g/L) anywhere in the mature biofilm obtained after 1000 h of simulation, the thickness was deemed too large and reduced. These simulations predicted that *S. aureus* could grow much thicker biofilms with *W*_max_ = 90 μm compared to *P. aeruginosa* with *W*_max_ = 30 μm.

**Fig. 2:**
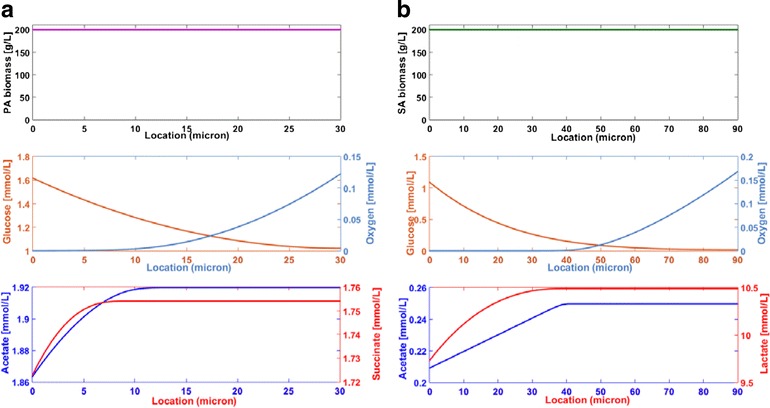
Spatially resolved predictions for single species biofilms. **a** *P. aeruginosa* with a maximum biofilm of thickness *W* = 30 μm. **b** *S. aureus* with a maximum biofilm of thickness *W* = 90 μm

Spatially resolved predictions obtained after 1000 h showed that the *P. aeruginosa* biofilm (Fig. 2a) were characterized by high biomass concentrations throughout the biofilm, low oxygen concentrations near the bottom of the biofilm furthest from the oxygen source, low glucose concentrations near the top of the biofilm furthest from the glucose source, and acetate and succinate as the primary metabolic byproducts. If the biofilm thickness was chosen larger than *W*_max_ = 30 μm, the *P. aeruginosa* biomass concentration dropped below 10 g/L at the bottom of the biofilm due to the combination of low oxygen penetration and very small anaerobic growth rates (see glucose results in Table 4). The *S. aureus* biofilm model predicted much deeper oxygen penetration (~50 μm vs. ~25 μm) due to more efficient use of oxygen for glucose oxidation (Fig. 2b). Superior anaerobic growth allowed *S. aureus* to produce much thicker biofilms under the same environmental conditions. Otherwise, the predictions were similar to those obtained with *P. aeruginosa* with lactate replacing succinate as a primary byproduct. If the *S. aureus* biofilm thickness was chosen larger than *W*_max_ = 90 μm, the biomass concentration dropped below 10 g/L near the top of the biofilm due to inadequate glucose penetration. The effect of the metabolite mass transfer coefficients on concentration gradients within the *S. aureus* biofilm are shown in Additional file 1: Figure S1.

### Metabolism of two species biofilms

Dynamic simulations were performed for two species biofilms consisting of *P. aeruginosa* and *S. aureus* using eight different hypothetical scenarios. Scenario 1 was the base case where the two bacteria competed for glucose and oxygen in the absence of byproduct crossfeeding, *P. aeruginosa* aerotaxis or pyocyanin-mediated lysis of *S. aureus*. We found the two species *W*_max_ = 80 μm, which was slightly less that the *S. aureus W*_max_ = 90 μm but substantially larger than the *P. aeruginosa W*_max_ = 30 μm. The two species *W*_max_ was a linear combination of the single species *W*_max_ values weighted by the average biomass concentrations in the two species biofilm:

7 $$ {W}_{\max }=\frac{\left(\mathsf{90}\mu \mathrm{m}\right)\left(\mathsf{165}\frac{\mathrm{g}}{\mathrm{L}}\right)+\left(\mathsf{30}\mu \mathrm{m}\right)\left(\mathsf{35}\frac{\mathrm{g}}{\mathrm{L}}\right)}{\left(\mathsf{165}+\mathsf{35}\right)\frac{\mathrm{g}}{\mathrm{L}}}=\mathsf{79.5}\mu \mathrm{m} $$

If the two species biofilm thickness was chosen larger than *W*_max_ = 80 μm, the biomass concentration dropped below 10 g/L near the top of the biofilm due to inadequate glucose penetration. The relationship between the two species was interpreted to be antagonistic with the incorporation of *P. aeruginosa* into a *S. aureus* biofilm reducing the achievable thickness for a given set of conditions.

**Fig. 3:**
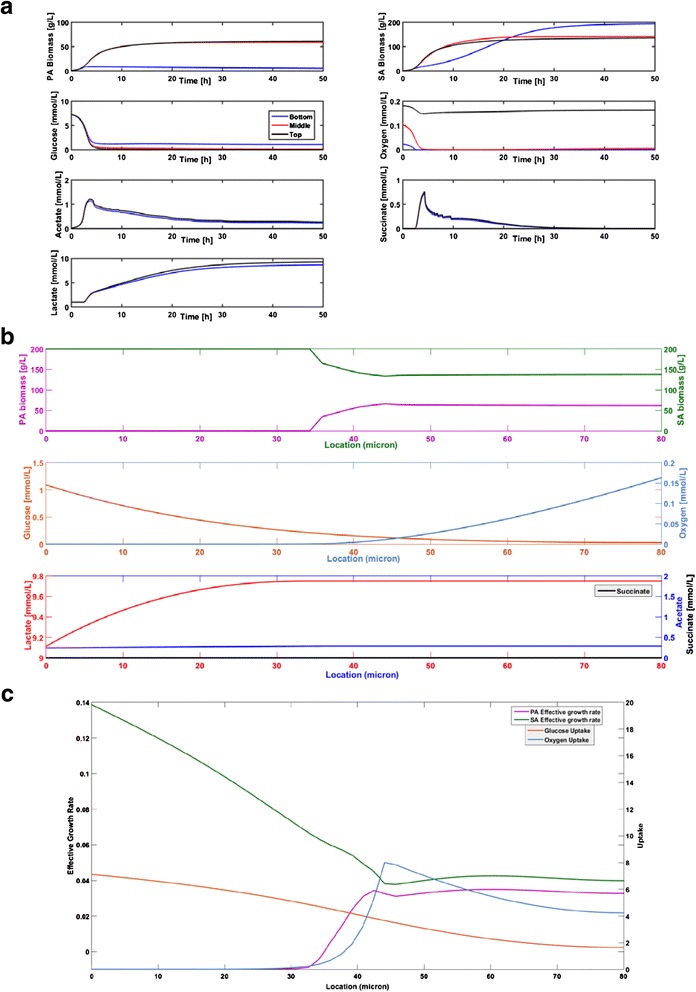
Predictions for a two species biofilm of thickness *W* = 80 μm (Base case scenario). **a** Time resolved predictions over the first 50 h at the bottom, middle and top of the biofilm. **b** Spatially resolved biomass and metabolite concentration predictions after 1000 h. **c** Spatially resolved effective growth and uptake rate predictions after 10 h

When the two species biofilm thickness was set equal to *W*_max_ = 80 μm, pseudo steady-state solutions were obtained after only 50 h of simulation (Fig. 3a). Oxygen was quickly depleted throughout most of the biofilm, except near the biofilm-air interface where an aerobic region was established as observed experimentally [81]. Similarly, glucose was rapidly depleted in all regions except near the tissue-biofilm interface where a glucose rich region was maintained. *S. aureus* was predicted to quickly establish dominance throughout the biofilm due to its higher local growth rates, especially in the anaerobic region. Initially acetate and succinate levels increased but thereafter they were predicted to decrease due to metabolite removal at the tissue-biofilm boundary. Lactate levels were predicted to remain high throughout the biofilm due to *S. aureus* synthesis in the anaerobic region and diffusion into the aerobic region. As in the single species biofilms, multispecies biofilm spatial profiles obtained after 1000 h of simulation (Fig. 3b) were characterized by the presence of a glucose rich, anaerobic region near the tissue-biofilm interface and a glucose depleted, aerobic region near the biofilm-air interface. *S. aureus* was predicted to be dominant throughout the biofilm, especially in the anaerobic region, while *P. aeruginosa* was predicted to be present only in the aerobic region. Byproduct profiles were similar to those obtained for the *S. aureus* single species biofilm (see Fig. 2b) with high lactate levels, low acetate levels and no succinate production. We attributed this behavior to partitioning of *P. aeruginosa* to the aerobic region where the synthesis of byproducts was substantially reduced.

**Fig. 4:**
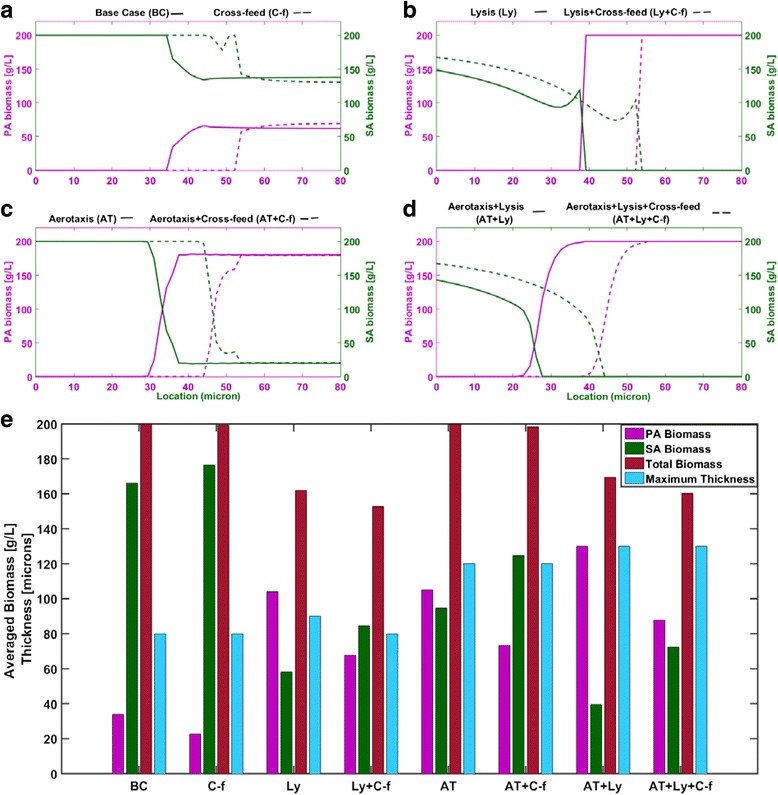
Predictions after 1000 h for two species biofilms of thickness *W* = 80 μm with different species interaction mechanisms. Base case (BC): competition for the nutrients glucose and oxygen. Cross-feed (C-f): nutrient competition plus cross feeding of lactate, succinate and acetate. Lysis (Ly): nutrient competition plus *P. aeruginosa* mediated lysis of *S. aureus*. Aerotaxis (AT): nutrient competition plus *P. aeruginosa* chemotaxis towards oxygen. **a**-**d** Spatially resolved biomass concentrations and **e** *P. aeruginosa* (PA), *S. aureus* (SA), total biomass concentrations averaged across the biofilm and maximum thickness for the eight considered scenarios

To further analyze how multispecies metabolism depended on position in the biofilm, local effective growth rates and nutrient uptake rates were determined from the base case (BC) simulation data. For species *i*, the local effective growth rate was calculated as the difference between the biomass restricted growth rate μ_*i*_(1-*Z*_*i*_/*Z*_max_) and the energy associated death rate *k*_*di*_ at a given position *z*. Consequently, the effective growth rate could be negative in nutrient lean regions. The calculations were performed using data collected at *t* = 10 h because these initial rates offered insights into biofilm physiology. *S. aureus* growth rates exceeded *P. aeruginosa* growth rates at all positions, especially in the anaerobic region near the bottom of the biofilm where *P. aeruginosa* death was predicted (Fig. 3c). Both species were predicted to have constant growth rates in the upper aerobic region. The *P. aeruginosa* growth rate decreased rapidly in the lower half due to decreasing oxygen availability such that death occurred in the first 35 μm. By contrast, the *S. aureus* growth rate increased rapidly in this region due to the increasing availability of glucose to support anaerobic growth. As time progressed, these local growth rates resulted in *S. aureus* dominance throughout the biofilm and *P. aeruginosa* presence only in the aerobic region (Fig. 4a). The glucose uptake rate increased monotonically from bottom to top of the biofilm, while the oxygen uptake rate was predicted to exhibit a maximum near the center because that location offered the optimal combined availability of glucose and oxygen to support consortium growth.

We also performed simulations assuming additional oxygen was available at the biofilm-tissue interface at a blood plasma concentration of 0.05 mmol/L [81]. This oxygen source allowed *P. aeruginosa* growth near the biofilm-tissue interface and therefore reduced spatial partitioning of the two species (Additional file 2: Figure S2). However, *S. aureus* still dominated throughout the biofilm due to it higher aerobic and anaerobic growth rates.

### Byproduct cross feeding

We hypothesized that cross feeding of secreted metabolic byproducts (lactate, succinate, acetate) would enhance the competitiveness of *P. aeruginosa* in the aerobic portion of the biofilm. Except for the inability of *S. aureus* to consume acetate, experimental studies as well as our FBA results (see Table 4) show that the two species are capable of metabolizing these byproducts in the presence of sufficient oxygen. Therefore, we investigated the impact of putative cross feeding by allowing each species to enhance its growth through uptake of the three byproducts (C-f scenario). The two species were assumed to uptake each byproduct with the same kinetics (see Table 2) due to lack of data on species and substrate specific uptake parameters.

The cross-feed scenario model predicted that *W*_max_ remained 80 μm when byproduct cross feeding was incorporated. Because glucose was the more energetically favorable carbon source, most of the available oxygen was used for glucose oxidation and little oxygen remained for lactate oxidation. Contrary to our hypothesis, cross-feeding reduced the region where *P. aeruginosa* was present and did not substantially increase the *P. aeruginosa* biomass concentration within this region (Fig. 4a). To succinctly quantify this behavior, the *P. aeruginosa* and *S. aureus* biomass concentrations were averaged across the biofilm and compared to average concentrations obtained for the base case scenario without cross feeding. While the total biomass concentration was not affected, cross-feeding increased the fraction of *S. aureus* relative to *P. aeruginosa* (Fig. 4e). Since lactate was the primary byproduct of the two species biofilm, we attributed this behavior to *S. aureus* having more efficient lactate metabolism. Single species FBA results show that *S. aureus* has higher growth rates on lactate under oxygen sufficient and oxygen limited conditions (see Table 4). Because similar behavior was observed for glucose metabolism in single species biofilms, the addition of lactate consumption was predicted to further increased *S. aureus* dominance in the aerobic region where sufficient oxygen was available for lactate oxidation.

### P. aeruginosa inhibition of S. aureus

*P. aeruginosa* is known to secrete a number of small molecules including pyocyanin which inhibit and lyse competing bacteria such as *S. aureus*. Additional simulations were performed to explore the impact of a putative pyocyanin-mediated lysis mechanism on the two species biofilm. When this mechanism was combined with nutrient competition (Ly scenario), the model predicted that *W*_max_ was slightly increased to 90 μm. Reduction of *S. aureus* biomass in the anaerobic region resulted in slightly higher glucose levels throughout the biofilm, allowing increased *P. aeruginosa* growth in the upper aerobic region and a greater biofilm thickness.

**Fig. 5:**
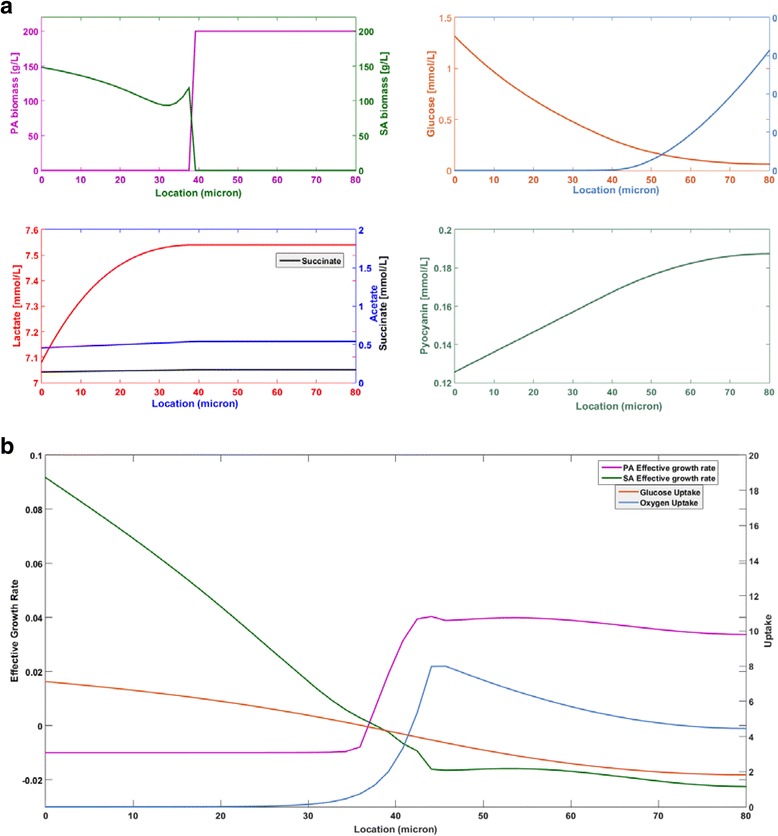
Spatially resolved predictions after 1000 h for a two species biofilm of thickness *W* = 80 μm with *pyocyanin mediated lysis of S. aureus*. **a** Biomass and metabolite concentration predictions after 1000 h. **b** Effective growth and uptake rate predictions after 10 h

To allow direct comparison with the other species interaction scenarios, simulations also were performed for an 80 μm thick biofilm. Spatial profiles showed sharp partitioning of the two species with *P. aeruginosa* dominant in the upper aerobic region of the biofilm (Fig. 5a). This effect was achieved at the expense of the *S. aureus* biomass concentration, which was substantially reduced in the lower anaerobic region and dropped to zero at 40 μm. While the oxygen profile was largely unaffected, reduction of *S. aureus* biomass in the anaerobic region resulted in slightly higher glucose levels throughout the biofilm. Pyocyanin levels were highest in the aerobic region due to diffusion and removal at the tissue-biofilm interface. The metabolic burden of synthesizing pyocyanin was predicted to have a minimal effect on *P. aeruginosa* growth due to the small enforced bound of 0.1 mmol/gDW/h. At a maximum glucose uptake rate of 10 mmol/gDW/h, only 2.2 % of available carbon was used for pyocyanin synthesis.

As before, local effective growth rates and nutrient uptake rates were calculated at *t* = 10 h to investigate how pyocyanin affected multispecies metabolism as a function of position in the biofilm. In this case, the effective local growth rate of *S. aureus* was calculated by subtracting the maintenance energy associated death rate and the pyocyanin associated death rate from the biomass restricted growth rate at a given point *z* (see Eq. (2)). In the upper aerobic region, pyocyanin release caused the *S. aureus* death rate to exceed the growth rate (Fig. 5b) such that *S. aureus* was eventually eliminated from this region. This balance was reversed in the anaerobic region, although *S. aureus* growth was substantially reduced. By contrast, the *P. aeruginosa* growth rate was very similar to the pyocyanin-free case (see Fig. 3c). The long-term effects of these local growth rates were a sharp spatial partitioning of the two species and reduced *S. aureus* biomass concentrations in the anaerobic region. As a result, slightly higher glucose uptake rates were predicted across the biofilm while the oxygen uptake rates were similar to the pyocyanin-free case (see Fig. 3c). When biomass concentrations were averaged across the biofilm, the pyocyanin-mediated lysis mechanism was predicted to substantially increase *P. aeruginosa* competitiveness at the expense of both *S. aureus* and total biomass (Fig. 4e).

Combining byproduct cross feeding and the lysis mechanism (Ly + C-f scenario) did not change *W*_max_ from the pyocyanin-free case (C-f scenario) but did increase *S. aureus* competitiveness by shifting the location where the species partitioned approximately 15 μm towards the biofilm-air interface (Fig. 4b). The addition of cross feeding resulted in average biomass concentrations that were roughly equal, while total biomass was reduced (Fig. 4e). We hypothesized that this unexpected effect was due to increased oxygen utilization by *S. aureus* for lactate oxidation. Although *S. aureus* biomass was simultaneously reduced by pyocyanin- mediated lysis, the oxygen used for *S. aureus* growth was not available for *P. aeruginosa* oxidative growth and total biomass decreased. Therefore, the pyocyanin mechanism was interpreted as an antagonistic mechanism by which *P. aeruginosa* increased its own competitiveness.

### P. aeruginosa aerotaxis

*P. aeruginosa* has a single flagellum that may allow motility in complex, heterogeneous environments such as biofilms [82] while *S. aureus* is generally viewed as non-motile [83]. More specifically, *P. aeruginosa* has been observed to chemotax towards higher oxygen environments, a process known as aerotaxis, which offer more favorable growth conditions [37]. To explore the impact of this putative aerotaxis mechanism on two species biofilm metabolism, the *P. aeruginosa* biomass equation included a chemotaxis term (see Eq. 2) and simulations were performed with both nutrient competition and aerotaxis (AT scenario). The energy requirements for chemotaxis were assumed negligible compared to growth. When aerotaxis was combined with nutrient competition, the model predicted that *W*_max_ was increased to 120 μm. Aerotaxis increased spatial partitioning of the two species (see below) such that *P. aeruginosa* had access to more oxygen for lactate respiration, resulting in a thicker biofilm.

**Fig. 6:**
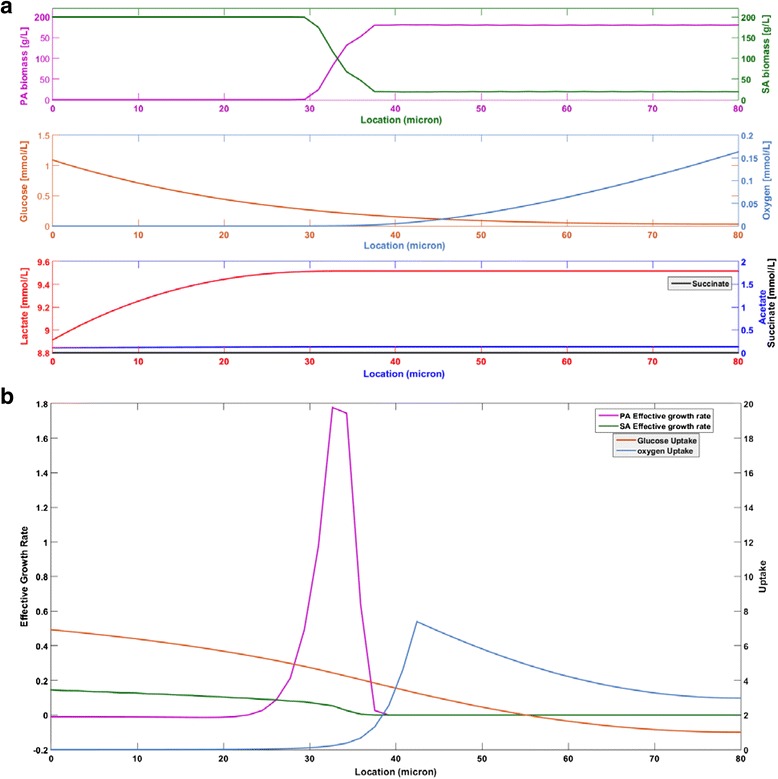
Spatially resolved predictions after 1000 h for a two species biofilm of thickness W = 80 µm with *P. aeruginosa* aerotaxis. **a** Biomass and metabolite concentration predictions after 1000 h. **b** Effective growth and uptake rate predictions after 10 h

Spatial profiles generated for an 80 μm biofilm show almost complete partitioning of the two species, with *P. aeruginosa* dominating in the upper aerobic half, only *S. aureus* present in the lower anaerobic region, and the two species coexisting for about 10 μm near the middle of the biofilm (Fig. 6). Aerotaxis allowed *P. aeruginosa* to substantially improve its competitiveness by increasing its concentration in the upper portion of the biofilm rather than by moving the transition region between the two species (see Fig. 3a). When averaged across the biofilm, the biomass concentrations of the two species were approximately equal while total biomass was unaffected compared to the aerotaxis-free case (Fig. 4e). Unlike pyocyanin-mediated lysis, aerotaxis could be viewed as an antagonistic mechanism by which *P. aeruginosa* increased its own competitiveness without reducing total cell densities.

When byproduct cross feeding was added to *P. aeruginosa* aerotaxis and nutrient competition (AT + C-f scenario), the model predicted *W*_max_ = 120 μm, the same value obtained in the absence of cross feeding. When simulations were performed for an 80 μm thick biofilm, the addition of cross feeding substantially increased *S. aureus* biomass in the biofilm while having a small negative impact on total biomass (Fig. 4e). Of the eight scenarios investigated, a maximum *W*_max_ = 130 μm was predicted when nutrient competition and aerotaxis were combined with pyocyanin-mediated lysis of *S. aureus* (AT + Ly scenario). In this case, *P. aeruginosa* had access to increased glucose due to *S. aureus* death in the anaerobic region and increased lactate due to the absence of *S. aureus* in the aerobic region, which combined to enhance *P. aeruginosa* growth and allow a thicker biofilm. For a nominal biofilm thickness of 80 μm, only *P. aeruginosa* was present in the upper 50 μm of the biofilm and the amount of *S. aureus* was relatively small in the lower anaerobic region (Fig. 4d). The further addition of byproduct cross feeding did not affect *W*_max_ but did increase *S. aureus* competitiveness (AT + Ly + C-f scenario) (Fig. 4e). Collectively, these predictions suggest that both pyocyanin-mediated lysis and aerotaxis are potentially powerful mechanisms for *P. aeruginosa* to enhance its competitiveness in multispecies biofilms with the faster growing facultative anaerobe *S. aureus*.

## Discussion

We hypothesized that the tendency of bacteria to self-organize into complex biofilm structures is at least partially driven by the appearance of metabolic niches [2]. To test this hypothesis, we developed spatiotemporal metabolic models of chronic wound biofilms consisting of the aerobe *Pseudomonas aeruginosa* and/or the facultative anaerobe *Staphylococcus aureus*. The models were comprised of genome-scale metabolic reconstructions combined with partial differential equations (PDEs) for the diffusion and consumption/synthesis of nutrients/byproducts within the biofilm. Both single species and two species biofilms were simulated by supplying glucose at the tissue-biofilm interface (bottom of the biofilm) and oxygen at the biofilm-air interface (top of the biofilm).

Simulations of single species biofilms were performed to compare the metabolic behavior of each species and to provide a basis for comparing the two species biofilm simulations. Although the biofilm thickness was specified a priori for each simulation, we gained insights into the collective biofilm substrate utilization efficiency as quantified by the maximum achievable thickness *W*_max_ under the specified environmental conditions. By assuming that the thickness was too large if the biomass concentration dropped below 10 g/L (5 % of the maximum value of 200 g/L) anywhere in the mature biofilm, the models predicted that *S. aureus* could grow much thicker biofilms with *W*_max_ = 90 μm compared to *P. aeruginosa* with *W*_max_ = 30 μm. Other differences predicted by the single species models were that *S. aureus* allowed much deeper oxygen penetration and produced much larger amounts of its primary metabolic byproduct lactate, a putative cross-fed metabolite. We attributed these differences in biofilm phenotypes to *S. aureus* having more efficient oxygen utilization in the upper aerobic region as well as substanially high growth rates in the lower anaerobic region. These model predictions could be experimentally tested by measuring metabolite concentration profiles using spatially resolved metabolomics [84–86] and gene expression profiles using spatially revolved transcriptomics [87, 88].

Two species biofilm simulations were performed for eight different scenarios to investigate the impact of putative mechanisms for interspecies interactions. For the base case involving only competition for the two nutrients glucose and oxygen, we found a maximum thickness *W*_max_ = 80 μm for the two species biofilm. This value represented a linear combination of the single species *W*_max_ values weighted by the average biomass concentrations in the two species biofilm. Dynamic simulations for an 80 μm thickness predicted that the two species would form a nearly mature biofilm in approximately 50 h, the time frame of many experimental studies [89, 90]. Two metabolic niches were quickly established in the simulated biofilm: an anoxic, glucose rich region in the lower half and an aerobic, glucose lean region in the upper half. Due to its superior anaerobic growth and more efficient oxygen utilization, *S. aureus* quickly established dominance throughout the biofilm. Steady-state spatial profiles demonstrated that this short-term behavior was maintained over long time periods, with *P. aeruginosa* only present in the aerobic region and lactate synthesized by *S. aureus* in the anaerobic region being the main byproduct. The experimental determination of spatially resolved biomass concentrations [91] would be beneficial in this context.

Cross feeding of secreted metabolic byproducts is common in bacterial communities [92, 93] and multispecies biofilms [94, 95]. For example, a cross feeding mechanism has been proposed for a polymicrobial infection system consisting of the two facultative anaerobes *Aggregatibacter actinomycetemcomitans* and *Streptococcus gordonii* [94]. To explore the hypothesis that cross feeding would enhance *P. aeruginosa* competitiveness (C-f scenario), we allowed each species to uptake all possible carbon sources (glucose, lactate, succinate, acetate) with the same uptake kinetics. Therefore, the results reflected differences in carbon metabolism efficiency and not differences in uptake properties. The model predicted that cross feeding would not increase *W*_max_ but would further enhance *S. aureus* competitiveness by reducing the region where *P. aeruginosa* was present. This prediction, which was consistent across all cross feeding scenarios investigated, was attributed to *S. aureus* having more efficient oxidative metabolism for the primary byproduct lactate. Byproduct cross feeding in *P. aeruginosa*/*S. aureus* chronic wound biofilms has not been experimentally studied to our knowledge and represents a promising area of research.

Cross feeding predictions were obtained assuming identical parameters for lactate uptake kinetics and non-growth associated ATP maintenance for the two species. Experimental studies with *P. aeruginosa* biofilms in the cystic fibrosis lung show that lactate actually can be a preferred carbon source to glucose [96, 97], suggesting enhanced lactate uptake capabilities. This environmental dependence emphasizes the importance of conducting uptake experiments and studying cross feeding under chronic wound relevant conditions. Well controlled planktonic growth experiments are needed to accurately estimate ATP maintenance demands of the two species, since lactate oxidation might confer a growth advantage to *P. aeruginosa* if the energetics are more favorable than those modeled.

*P. aeruginosa* secretes a wide variety of inhibitory compounds that have been shown to enhance its competitiveness against competing bacteria in multispecies biofilm communities [98–100]. To quantify the impact of a representative small molecule inhibitor, *P. aeruginosa* was forced to secrete pyocyanin and *S. aureus* was lysed by pyocyanin diffusing from high concentration regions. When combined with nutrient competition, the pyocyanin-mediated lysis mechanism produced a slightly larger *W*_max_ = 90 μm and substantially increased *P. aeruginosa* competitiveness by inducing *S. aureus* death in the aerobic region. While we tuned our model to obtain reasonable extracellular pyocyanin concentrations [71], key parameters (i.e. synthesis rate, diffusion coefficient, killing rate) associated with the mechanism are unknown and need to be experimentally determined to accurately quantify the effect in chronic wound environments.

The motility of *P. aeruginosa* allows coordinated movement of cells towards higher oxygen levels through a chemotactic response known as aerotaxis [37]. When putative *P. aeruginosa* aerotaxis was combined with nutrient competition (AT scenario), our biofilm model predicted substantially thicker biofilms (*W*_*max*_ = 120 μm) and nearly complete species partitioning as observed experimentally [47, 101], with *P. aeruginosa* dominant in the upper aerobic half and only *S. aureus* present in the lower anaerobic region. While not producing as dramatic increase in the *P. aeruginosa*/*S. aureus* ratio as the pyocyanin mechanism, aerotaxis did not negatively impact total biomass within the biofilm. Therefore, we interpreted aerotaxis as a less antagonistic mechanism than pyocyanin secretion by which *P. aeruginosa* could enhance its own competitiveness. However, *P. aeruginosa* aerotaxis within biofilm environments has not been demonstrated to our knowledge. Therefore, biofilm reactor experiments aimed at demonstrating and quantifying the aerotactic response would be highly beneficial. Experimental testing could be achieved through a combination of traditional and spatially resolved omics technologies [86, 87, 102].

## Conclusions

Chronic wounds are often colonized by bacteria consortia growing as biofilms on a complex mixture of wound exudate. Improved understanding of these complex multispecies systems is required to develop more rational and effective antibiotic therapies for biofilm eradication. We developed genome-scale spatiotemporal models of a two species consortium comprised of the chronic wound isolates *Pseudomonas aeruginosa* and *Staphylococcus aureus* to investigate the impact of putative species interaction mechanisms on biofilm physiology. The models were used to analyze the metabolic differences between single species and two species biofilms and to investigate the impact of nutrient competition, byproduct cross feeding, *P. aeruginosa* inhibition of *S. aureus* growth and *P. aeruginosa* aerotaxis on the relative abundance and spatial distribution of each species. The key predictions of our computational modeling study were:

1. The two species system was predicted to support a maximum biofilm thickness much greater than *P. aeruginosa* alone but slightly less than *S. aureus* alone, suggesting an antagonistic metabolic effect of *P. aeruginosa* on *S. aureus*.
2. Nutrient gradients imposed by supplying glucose at the bottom and oxygen at the top of the biofilm induced spatial partitioning of the two species, with *S. aureus* most concentrated in the lower anaerobic region and *P. aeruginosa* present only in the upper aerobic region.
3. When each species was allowed to enhance its growth through consumption of secreted metabolic byproducts assuming identical uptake kinetics, the competitiveness of *S. aureus* was further enhanced due to its more efficient lactate oxidative metabolism.
4. Lysis of *S. aureus* by the small molecule inhibitor pyocyanin secreted from *P. aeruginosa* and/or *P. aeruginosa* aerotaxis towards high oxygen levels were predicted to enhance spatial portioning of the two species and to increase *P. aeruginosa* competitiveness in the aerobic region.

These model predictions require further validation through the execution of targeted experiments that augment existing results in the literature that support our conclusions. We believe that *P. aeruginosa* lysis of *S. aureus* combined with nutrient competition is a particularly relevant scenario for which model predictions could be tested experimentally.

## Additional files

Additional file 1: Figure S1. Spatially resolved byproduct (acetate, lactate) concentration predictions after 1000 h for a two species biofilm of thickness *W* = 80 μm with three different values (1 × 10^−4^ cm/s, 2 × 10^−4^ cm/s, 4 × 10^−4^ cm/s) of the metabolite mass transfer coefficient. (DOCX 61 kb) Additional file 2: Figure S2. Spatially resolved predictions after 1000 h for a two species biofilm of thickness *W* = 80 μm when oxygen was supplied from both ends of the biofilm. Oxygen was supplied at the tissue-biofilm interface at a blood plasma concentration of 0.05 mmol/L. Oxygen was supplied at the biofilm-air interface at a concentration of 0.21 mmol/L for an aqueous solution in equilibrium with atmospheric oxygen. The effect of oxygen from the blood plasma on biofilm species organization is studied. The species partitioning is obeserved between W = 18–32 μm and two species coexist in rest of the biofilm. (DOCX 71 kb)

## Abbreviations

DFBAlab: Dynamic flux balance analysis laboratory
FBA: Flux balance analysis
LP: Linear program
ODE: Ordinary differential equation
PDE: Partial differential equation
