# Supplementary figures and images for: Metabolic modeling of a chronic wound biofilm consortium predicts spatial partitioning of bacterial species
Source: BMC Syst Biol. 2016 Sep 7;10(1):90. doi: 10.1186/s12918-016-0334-8 (PMC5015247; doi:10.1186/s12918-016-0334-8)

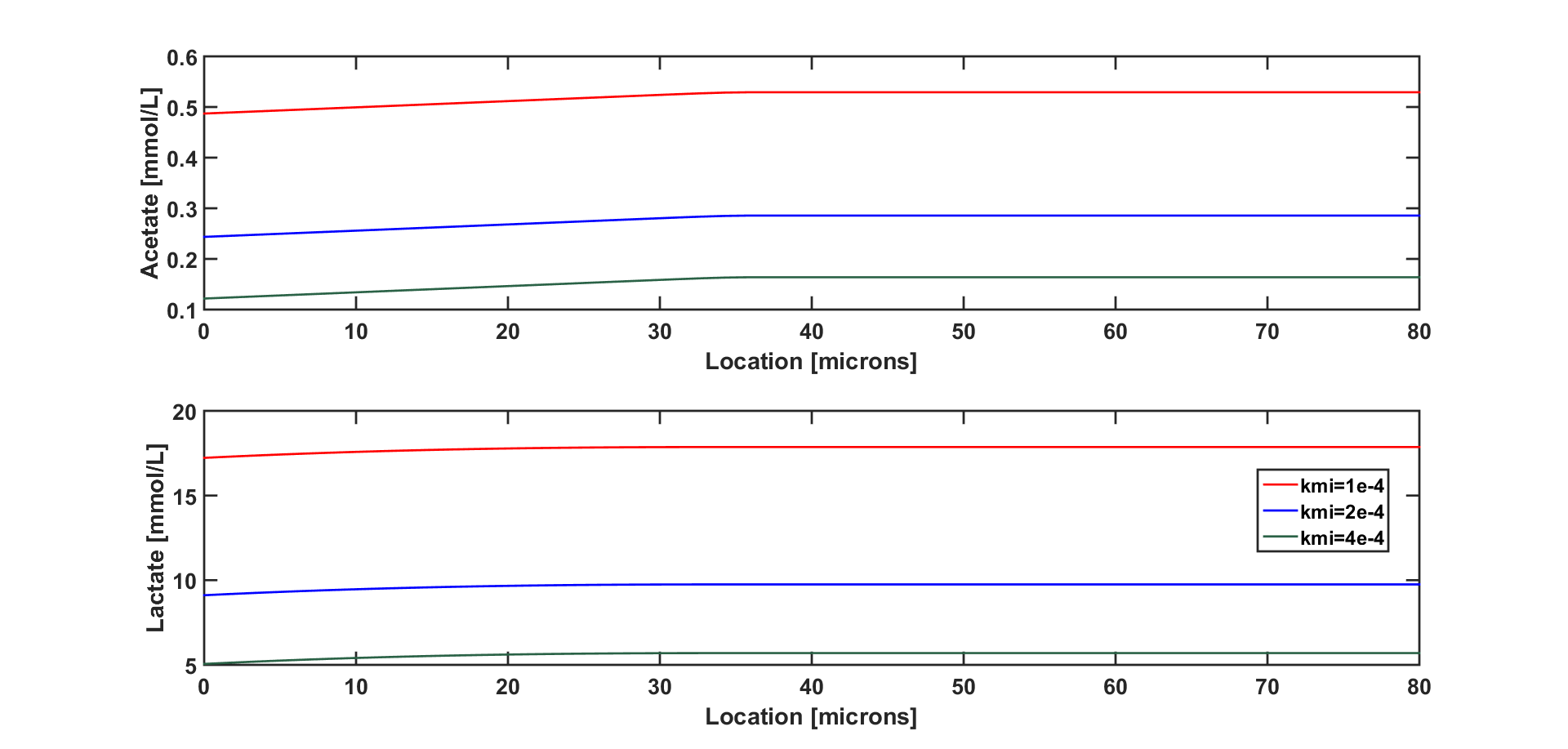

Supplement: Additional file 1: Figure S1. — Spatially resolved byproduct (acetate, lactate) concentration predictions after 1000 h for a two species biofilm of thickness W = 80 μm with three different values (1 × 10−4 cm/s, 2 × 10−4 cm/s, 4 × 10−4 cm/s) of the metabolite mass transfer coefficient. (DOCX 61 kb) [file 12918_2016_334_MOESM1_ESM.docx]

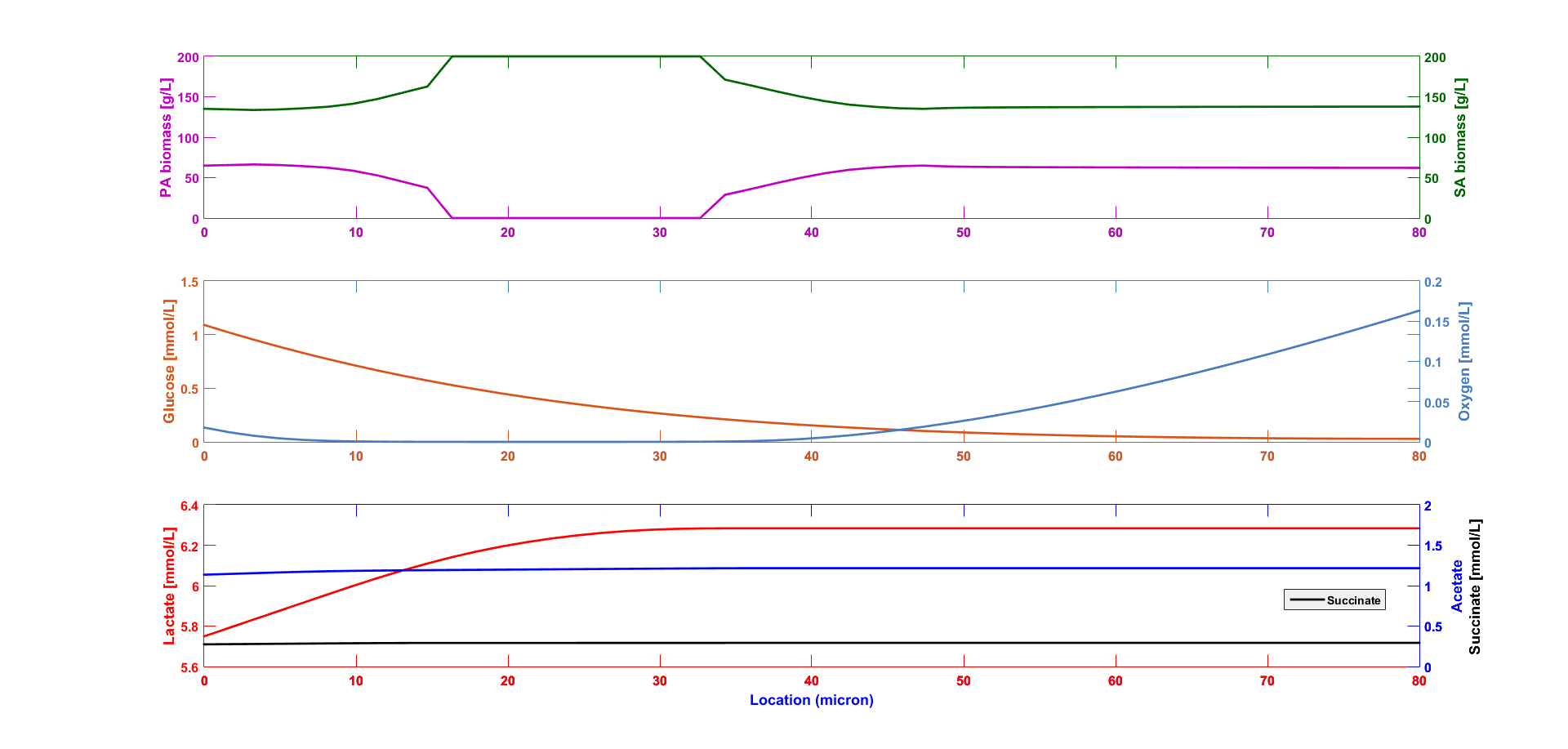

Supplement: Additional file 2: Figure S2. — Spatially resolved predictions after 1000 h for a two species biofilm of thickness W = 80 μm when oxygen was supplied from both ends of the biofilm. Oxygen was supplied at the tissue-biofilm interface at a blood plasma concentration of 0.05 mmol/L. Oxygen was supplied at the biofilm-air interface at a concentration of 0.21 mmol/L for an aqueous solution in equilibrium with atmospheric oxygen. The effect of oxygen from the blood plasma on biofilm species organization is studied. The species partitioning is obeserved between W = 18–32 μm and two species coexist in rest of the biofilm. (DOCX 71 kb) [file 12918_2016_334_MOESM2_ESM.docx]
